# Sodium-Glucose Cotransporter 2 Inhibitor-Induced Euglycemic Diabetic Ketoacidosis: The Other Side of the Coin!

**DOI:** 10.7759/cureus.58341

**Published:** 2024-04-15

**Authors:** Sangita D Kamath, Umesh Kumar, Vikki Shrivastava

**Affiliations:** 1 Internal Medicine, Tata Main Hospital, Jamshedpur, IND; 2 General Medicine, Tata Main Hospital, Jamshedpur, IND

**Keywords:** dehydration, medication, euglycemia, diabetic ketoacidosis, sglt2 inhibitors

## Abstract

Euglycemic diabetic ketoacidosis (EDKA) though rare is a life-threatening complication of sodium-glucose cotransporter 2 (SGLT2) inhibitors. With their increasing use in the management of type 2 diabetes mellitus (T2DM) due to long-term beneficial effects, the incidence of this complication is on the rise. We report a case of a 58-year-old lady with a history of T2DM on multiple anti-diabetes medications including dapagliflozin for one year, who during intercurrent illness developed EDKA. Her blood sugar on admission was 203 mg/dL, and arterial blood gas showed high anion-gap metabolic acidosis (HAGMA) with ketonuria and ketonemia (blood beta-hydroxybutyric (BOHB) acid level: 5.4 mmol/L). Low carbohydrate intake, dehydration resulting from repeated vomiting, and skipping the previous two days’ dose of insulin could have precipitated this condition. She was treated with intravenous fluids, insulin, 5% dextrose infusion, and potassium supplements with complete resolution of acidosis after about 90 hours. This case signifies the importance of awareness of the link between the use of SGLT2 inhibitors and EDKA and early recognition of this complication to reduce morbidity and mortality. Furthermore, it also emphasizes the need for clinicians to educate their patients taking these drugs to stop them during the intercurrent illness to prevent them from developing EDKA.

## Introduction

SGLT2 inhibitors, also known as gliflozins, were approved by the US Food and Drug Administration (FDA) in 2013 for the treatment of type 2 diabetes mellitus (T2DM) [1]. They act through the inhibition of SGLT2 transmembrane protein (receptor) expressed on the apical cells of proximal convoluted tubules (PCT), which results in the inhibition of glucose reabsorption in PCT and facilitates its excretion in urine [1]. In addition to their blood glucose lowering effect, they have cardiovascular and reno-protective effects and cause modest reductions in blood pressure and weight loss. From anti-diabetes drugs, their scope of use is now expanded to the treatment of heart failure (HF) with reduced ejection fraction (HFrEF) and with preserved ejection fraction (HFpEF) regardless of the presence of diabetes. Thus, due to their undisputable beneficial effects, their use has gained popularity in the recent past. However, the European Medicines Agency and US FDA have cautioned against the life-threatening complication of euglycemic diabetic ketoacidosis (EDKA), based on the incidents identified through the adverse reporting event system [2,3]. EDKA is defined by a triad of high anion-gap metabolic acidosis (HAGMA) with high serum and urine ketones and a blood glucose level of less than 250 mg/dL [1]. The possibility of conventional DKA is often thought of in an appropriate clinical context in a patient of T2DM, but that of EDKA is rarely considered or is missed, in view of normal or near-normal blood glucose levels. We, therefore, report this case to create awareness among clinicians as we deep dive into its realms of diagnostic challenges and management.

## Case presentation

 A 58-year-old lady was admitted for multiple episodes of vomiting, abdominal pain with distension of three days duration associated with generalized weakness, and fever on and off for four days. She did not have addictions or allergies. She had a history of T2DM and hypertension for 10 years. She was presently on insulin Ryzodec (co-formulation of Aspart and Degludec) 20 units once daily subcutaneously, sitagliptin 100 mg once daily orally, metformin 500 mg twice daily, aspirin 75 mg once daily, atorvastatin 10 mg once daily, telmisartan 40 mg once daily, and dapagliflozin 10 mg once daily (started about one year back). On examination, she was coherent and had no pallor, icterus, or cyanosis but was dehydrated with dry oral mucosa, lips, and tongue. She was afebrile, with blood pressure of 112/70 mm Hg, pulse rate of 114/minute, and respiratory rate of 26/minute with accessories working. Examination of cardiovascular, respiratory, gastrointestinal, and neurological systems was unremarkable. Blood investigations were as inTable 1.

**Table 1:** Lab investigations (hematological and biochemical tests) Hb, hemoglobin; PT, prothrombin time; INR, international normalized ratio; MCV, mean corpuscular volume; CRP, C-reactive protein; ALT, alanine transaminase; AST, aspartate transaminase; ALP, alkaline phosphatase

Investigations	Values
Hb	12.5 gm/dL
Total leucocyte count	1.24x10^9^/L
MCV	90.6 fL
Platelet count	168x10^9^/L
CRP	4.1 mg/dL
Pro-calcitonin	0.9 ng/mL
Total serum proteins	7.6 g/dL
Serum albumin	4.2 g/dL
Serum globulin	3.4/dL
Serum creatinine	0.98 mg/dL
Total bilirubin	0.95 mg/dL
Direct	0.7 mg/dL
ALT	27.1 U/L
AST	34.8 U/L
ALP	140.8 U/L
PT (INR)	1.02
ESR (Westergren)	52 mm 1st hour
Serum lipase	68.2 U/L

Her glycated hemoglobin (HbA1c) level was 9.5%. Urine and stool routine examinations were normal. The blood culture did not reveal any microbial growth. Tests for malaria, dengue, and chikungunya were negative. Abdominal ultrasound showed normal study except for grade one fatty liver. Chest radiograph and echocardiography were normal. Her electrocardiography (ECG) revealed sinus tachycardia. Her random blood sugar on admission was 203 mg/dL. Arterial blood gas showed a pH of 7.142, PCo_2_ of 17.6 mmHg, PO_2_ of 162 mmHg, sodium (Na+) of 134 mmol/L, chloride (Cl^-^) of 109 mmol/L, potassium (K+) of 3.4 mmol/L, bicarbonate (HCO3^-^) of 9.0 mmol/L, and lactate of 1.1 mmol/L. Thus, ABG showed HAGMA with a base deficit of 16.6 mmol/L.

Differentials for HAGMA include ketoacidosis due to starvation, alcohol, and diabetes (DKA); lactic acidosis; acidosis due to acute and chronic renal failure; and poisoning by toxins like ethylene glycol, methanol, and salicylates [4]. Her calculated serum osmolality was 284 mOsm/Kg while the measured serum osmolality by osmometer was not checked, as this facility is not available in our hospital. Her urine ketone bodies by dipstick method was 4+ and her serum level of beta-hydroxybutyric (BOHB) acid was 5.4 mmol/L (normal<0.5 mmol/L) confirming the diagnosis of ketoacidosis. Furthermore, her serum lactate and creatinine levels were normal. There was no history of illicit drug abuse. As her blood sugar on presentation was 203 mg/dL, diagnosis of EDKA induced by dapagliflozin and precipitated by an infection was made. Dapagliflozin was stopped. She was given intravenous 0.9% normal saline (up to 2.5 L over three hours) to correct dehydration, infusion of short-acting, regular insulin (Actrapid) at 4 units/hour (0.1 units/kg/hour) with 5% dextrose at 100 mL/hour through a different peripheral line. Initially, blood glucose level was monitored every 2 hours for 24 hours and was maintained between 150 to 200 mg/dL. Her ABG was monitored every six hours. Glucose and insulin infusion were continued till the resolution of ketoacidosis by ABG and was further confirmed by measuring her blood BOHB acid levels. It took about 90 hours for complete resolution of ketoacidosis. Broad spectrum antibiotic piperacillin-tazobactum 4.5 g intravenously was started in view of sepsis (as demonstrated by high procalcitonin and fever), though we could not demonstrate the exact cause of sepsis. On the fourth day, her repeat ABG showed a pH of 7.367 and HCO3^-^ of 23.2 mmol/L. Ketone bodies in urine were not detectable and blood BOHB acid level was 0.5 mmol/L. She was discharged on the sixth day in stable condition with advice to follow up in the out-patient department after a week. She was discharged on injection premix insulin (Mixtard 30/70) 12 and 8 units twice daily in addition to anti-hypertensive medication.

## Discussion

SGLT2 inhibitors are the recent class of oral anti-diabetes medication. Several clinical trials have demonstrated their beneficial effects on cardiovascular and renal outcomes, irrespective of their blood glucose-lowering potential [5,6]. Given their overall risk-benefit ratio, their use is recommended by the US FDA as the second-line drugs after metformin for control of glycemic status and as per American Diabetes Association (ADA) 2022 guidelines, as first-line pharmacological therapy for T2DM (usually together with metformin), especially in patients with chronic kidney disease (CKD), stroke, or HF [7].

However, in 2015, the FDA cautioned that their use is associated with serious adverse drug events of EDKA [1]. Increased risk is observed with all SGLT-2 inhibitors suggesting class effect, with canagliflozin (hazard ratio: 3.58) having the highest risk [8]. Retrospective analysis of these cases revealed that some of these patients actually had type 1 diabetes mellitus (T1DM) and latent autoimmune diabetes of adults (LADA) and were misdiagnosed as T2DM [9,10,11]. The median time for the development of EDKA after starting SGLT2 inhibitors was two weeks (range one to 175 days) and in 50% of these cases, there was a precipitating factor like insulin deficiency [11]. The incidence of EDKA as reported by Goldenberg was 0.16 to 0.76 events per 1000 patient-years, but with increasing use of SGLT2 inhibitors, its incidence is likely to go up further [8].

The proposed mechanism for the development of EDKA is the switch from carbohydrate to fat metabolism due to carbohydrate depletion secondary to renal glycosuria, leading to low serum glucose level, decreased insulin level (insulinopenia), and increased glucagon release from pancreatic alpha cells. The resulting lower insulin glucagon ratio stimulates lipolysis, augments free fatty acid (FFA) delivery to the liver, and their beta-oxidation, and, thus, promotes ketogenesis in the liver. Furthermore, they also increase renal absorption of ketone bodies. Glycosuria leads to normal or near-normal blood glucose levels [1]. This condition can be precipitated by any intercurrent illness, post-surgery, sepsis, alcoholism, and use of a ketogenic diet, where decreased intake of carbohydrates combined with lower glucose levels can further suppress insulin release and lead to EDKA. Other causes of EDKA include chronic alcoholism, pregnancy, glycogen storage disorders, chronic liver disease, and prolonged starvation [12,13]. Clinically, it presents like conventional DKA with abdominal pain, nausea, vomiting, breathlessness, and dehydration.

Most cases published in the literature have reported early development of EDKA (up to a maximum of 25 weeks of initiation of the drug) [11]. However, our case was unique in the sense that the patient was on dapagliflozin for up to one year. Fever and decreased carbohydrate intake due to vomiting might have precipitated EDKA in our patient. Sood M et al. described a case of EDKA in a diabetic patient who was on the Atkins diet for weight loss and was also prescribed canagliflozin [14]. Hayami T et al. reported a similar case in a 32-year-old diabetic with Prader-Willi syndrome who was prescribed a low carbohydrate diet and developed euglycemic DKA after starting ipragliflozin 13 days prior [15]. Diaz-Ramos A et al. described a case of a 44-year-old female who presented with EDKA who was started on canagliflozin four weeks earlier and had stopped insulin for two weeks [16]. Peterson C et al. described EDKA in a 28-year-old type 2 diabetic, five days after he was initiated on empagliflozin 10 mg post-percutaneous coronary intervention (PCI) and required 120 hours of continuous glucose-insulin infusion for closure of the AG [17]. In a systematic review by Dutta et al., common risk factors for the development of EDKA in patients on SGLT2 inhibitors were female sex, surgery in the recent past, and concomitant use of canagliflozin with metformin [18]. Pujara S et al. described a case of dapagliflozin-induced EDKA, where glycosuria and ketonemia persisted for 10 days despite stopping the drug [19]. Finucane et al. proposed the presence of a rare variant of SGLT receptor that could have more than usual affinity for SGLT2 inhibitor ligand, resulting in SGLT2 inhibitor-associated EDKA [20].

The half-life of SGLT2 inhibitors is prolonged, ranging from 11 to 13 hours. Hence, it is recommended by the FDA to stop these drugs for five half-lives for 97% of the drug to get eliminated from the body, that is 72 hours prior to an elective surgery and also during extremely stressful physical activity (e.g., running a marathon) [21]. Patients of renal failure may take up to eleven half-lives for drug elimination, requiring earlier stoppage of the drug [22]. The drug should be stopped immediately once the condition is suspected. It may be restarted when there is complete clearance of ketoacidosis, and the precipitating factor is addressed. In case of recurrence, the drug should be permanently stopped. The drugs should be withheld in the post-operative period till the patient resumes normal diet and hydration.

The management of EDKA is almost like that of DKA, as far as correction of dehydration and potassium supplementation are concerned. Dehydration in EDKA is usually not as severe as in DKA. However, patients of EDKA require both dextrose and insulin infusion simultaneously for providing and utilizing metabolic fuel of glucose. The insulin needs to be given in an appropriate dose of 0.05-0.1 units/kg/hour. There is a tendency to give lesser insulin due to the low-normal blood glucose level and fear of causing hypoglycemia but this might prolong the course of the disease and delay its resolution. The dose is titrated to maintain blood glucose between 150 and 200 mg/dL. The endpoint of stopping infusion is the correction of metabolic acidosis and normalizing blood levels of BOHB acid.

Our case highlights the need for having a high index of suspicion and continuous vigilance for the development of this complication in patients on SGLT2 inhibitors. As patients with EDKA may have normal or near-normal blood sugar levels, the diagnosis may be overlooked, unless thought of. The blood glucose levels may be falsely reassuring for a clinician not aware of this dreaded complication. It is, hence, recommended to do an ABG analysis with a ketone body test to avoid the pitfall. A blood BOHB level assessment from the laboratory or point of care (POC) ketone meter will clinch the diagnosis. A blood BOHB level of more than 3 mmol/L is suggestive of EDKA and monitoring BOHB levels will also help in determining the response to therapy.

An additional learning in this case, which needs to be highlighted, is regarding their use during the intercurrent illness and post-operative period. Patients should be educated regarding “sick-day rules,” which suggest stopping these drugs to prevent the development of this complication [3]. Also, while prescribing this class of drugs, the physician should warn their patients about the potential risk factors, like fasting, dehydration, physical exertion, skipping insulin, and alcohol binge drinking, which may precipitate this complication and withhold these drugs during this period. During pre-anesthetic check-ups for any elective surgery, these patients have to be made aware of the development of this complication, despite stopping the drug.

## Conclusions

This case report adds to the existing evidence in the literature supporting the relationship between the use of SGLT2 inhibitors and EDKA. It may be very challenging to diagnose this condition in the absence of high blood glucose levels as seen in typical DKA. It is imperative to check blood gases and ketone body levels in patients on SGLT2 inhibitors as the diagnosis cannot be excluded based on blood glucose levels alone. Unexplained HAGMA even in the setting of normal blood glucose level should prompt the clinician to exclude this dreaded complication. This report also highlights the importance of the role of patient education on the “sick-day rules” before starting this class of drugs. Clinicians should have a high index of suspicion for early detection of this complication and avoid delay in treatment.
